# Utilizing the International Classification of Functioning, Disability and Health to Describe Quality‐of‐Life Assessment in Older Adults in a Middle‐Income Country

**DOI:** 10.1002/pri.70271

**Published:** 2026-06-30

**Authors:** Maik Berber Freitas, Gustavo Pietracatelli Janizello, Wallace Pereira Silva, Léia Cordeiro de Oliveira, Vívian Elaine Alflen Soares, Ariadne Cardoso da Silva, Bibiana Caldeira Monteiro, Cid André Fidelis de Paula Gomes, Soraia Micaela Silva

**Affiliations:** ^1^ Postgraduate Program in Rehabilitation Sciences Universidade Nove de Julho (UNINOVE) São Paulo São Paulo Brazil

**Keywords:** disability and health, health‐related quality of life, international classification of functioning, older adults

## Abstract

**Background and Purpose:**

The global aging population is rapidly increasing, prompting the United Nations to declare the “Decade of Healthy Aging” (2021–2030) to improve the quality of life for older adults. Health‐related quality of life (HRQoL) is crucial in this context, and the International Classification of Functioning, Disability and Health (ICF) provides a standardized framework for its evaluation. This study aimed to apply a previously proposed method for converting SF‐36 domain scores into ICF qualifiers in community‐dwelling older adults attending primary care services in a middle‐income country, describing the resulting classification of functioning domains using standardized ICF qualifiers.

**Methods:**

A cross‐sectional study was conducted with older adults aged 60 and above who accessed primary healthcare services and had no cognitive impairment. Participants underwent HRQoL assessment using the SF‐36, and ICF codes previously linked to SF‐36 domains were classified using ICF qualifiers. A simple calculation method was developed to convert SF‐36 scores into ICF qualifiers.

**Results:**

The study included 52 participants, with a mean age of 71.6 ± 7.0 years, 92.3% of whom were women. The ICF framework qualified 27 codes from SF‐36 domains. Moderate impairments were observed in “Bodily Pain,” “General Health,” and “Vitality” domains, while “Physical Functioning,” “Social Functioning,” and “Mental Health” domains showed mild impairments. No impairments were noted in the “Role Physical” and “Role Emotional” domains.

**Discussion:**

The application of ICF qualifiers to SF‐36 domain scores yielded standardized classifications of HRQoL domains across the ICF framework, allowing HRQoL outcomes to be described according to the severity levels defined by the ICF qualifier scale.

## 1 Introduction

Population aging is occurring at an accelerated pace, leading to a global demographic transition. According to United Nations projections, the number of older adults is expected to reach 1.6 billion by 2050, more than double the population recorded in 2021 (World Health Organization 2023a, 2023b). As part of a strategy for healthy aging, the United Nations General Assembly has declared the 2021–2030 period as the “Decade of Healthy Aging” (World Health Organization 2020). Initiatives developed during this Decade aim to change perceptions, attitudes, and behaviors related to aging; facilitate the participation of older adults in and their contributions to communities and society; provide integrated care and primary health services tailored to individual needs; and ensure access to long‐term care for older adults. The goal is to enhance the quality of life in the later years of life (World Health Organization 2020).

Health‐related quality of life (HRQoL) refers to an individual's perception of illness and its effects on physical, functional, emotional, and social well‐being (Franchignoni and Salaffi 2003; Santos et al. 2022). Krawczyk‐Suszek and Kleinrok (2022) investigated the relationship between sociodemographic factors and HRQoL in healthy individuals aged 65 and older adults in Poland. Using the 36‐Item Short Form Health Survey (SF‐36) questionnaire, the researchers assessed HRQoL across different age groups, considering variables such as sex, place of residence, education, smoking, and physical activity. The results indicated a decline in HRQoL with advancing age, particularly in those over 80 years old. The study emphasizes the importance of modifiable factors, such as physical activity, in maintaining HRQoL among older adults. This finding highlights the importance of monitoring HRQoL in older adults, even in the absence of diagnosed health conditions.

According to Cieza and Stucki (2005), the International Classification of Functioning, Disability, and Health (ICF) provides an excellent framework for comparing the content of HRQoL instruments, offering a standardized and universal language. The ICF allows for the comparison of the epidemiological situation of different population groups globally. Additionally, the ICF is a tool with significant potential to address various intersectoral demands, including public healthcare, social welfare, and social security systems. These characteristics make the ICF highly relevant to healthcare services for older adults. The concept of HRQoL is closely linked to the biopsychosocial model of the ICF (World Health Organization 2001).

In line with this, the WHO Rehabilitation 2030 initiative (Gimigliano and Negrini 2017; Cieza 2019) and the landmark World Health Assembly resolution on rehabilitation (“WHA Resolution”) (World Health Organization 2023a, 2023b) emphasize the importance of strengthening rehabilitation within health systems. In response to this global call, the Physical and Rehabilitation Medicine Section and Board of the European Union of Medical Specialists have been actively developing standards and tools based on functioning. This development is essential for advancing academic capacity in rehabilitation and addressing the growing global focus on the needs of an aging population.

The integration of ICF‐based assessments is particularly relevant for low‐ and middle‐income countries such as Brazil. Two contextual features support the relevance of this approach in such settings: the SF‐36 is a low‐cost, widely validated measure of health‐related quality of life with established psychometric properties in the Brazilian population (Laguardia et al. 2011), and Brazil has established policy frameworks supporting the use of the ICF, particularly in disability and rehabilitation contexts (Brasil 2015). Whether these contextual advantages translate into measurable gains in assessment quality or healthcare efficiency remains to be evaluated empirically.

Previous studies have demonstrated that specific ICF codes are associated with HRQoL tools (Fréz et al. 2014; Peres et al. 2018; Silva et al. 2019; Santos et al. 2022). These studies found that ICF qualifiers provide a comprehensive and standardized method for assessing HRQoL, enabling the quantification of the severity of identified issues (Fréz et al. 2014; Peres et al. 2018; Santos et al. 2022). To our knowledge, no prior study has applied a systematic SF‐36‐to‐ICF qualifier conversion approach specifically in community‐dwelling older adults, though linkage studies have been conducted in other clinical populations using related methods, including amputee patients (Fréz et al. 2014), caregivers of hospitalized patients (Peres et al. 2018), and stroke survivors (Silva et al. 2019; Santos et al. 2022).

The ICF provides a standardized framework for describing functioning and disability and may complement the interpretation of HRQoL outcomes. Using the ICF categories previously linked to the SF‐36 by Bernardelli et al. (2021) and the qualifier assignment approach proposed by Fréz et al. (2014), SF‐36 scores can be expressed according to the severity levels defined by the ICF. Therefore, the aim of this study was to apply this conversion approach to a sample of community‐dwelling older adults and describe HRQoL outcomes within the ICF framework.

## 2 Methods

### 2.1 Study Design

An observational, cross‐sectional study was conducted in a sample of older adults recruited from the outpatient clinics of Nove de Julho University.

### 2.2 Participants

Individuals aged 60 and older were selected through a non‐probabilistic convenience sampling method. The inclusion criteria were an age older than 60 years and who used at least one of the primary health care services at the outpatient clinics of Nove de Julho University. Individuals with cognitive impairments, as assessed by the Mini Mental State Examination (MMSE), were excluded from the study. This study received approval from the Human Research Ethics Committee of Nove de Julho University, São Paulo, Brazil (CAAE: 50910721.9.0000.5511; approval number: 4.944.103).

### 2.3 Instruments

#### 2.3.1 36‐Item Short Form Health Survey (SF‐36)

The translation and adaptation of the Brazilian version of the SF‐36 were performed by Ciconelli et al. (1999). The measurement properties of the Brazilian version of the SF‐36 were assessed, and the authors concluded that the instrument was adequate and clinically useful.

The SF‐36 is a widely used instrument for assessing HRQoL and measures eight domains: “Physical Functioning”, “Role Physical”, “Bodily Pain”, “General Health”, “Vitality”, “Social Functioning”, “Role Emotional”, and “Mental Health” (Lins and Carvalho 2016). The final score ranged from 0 to 100 points, with lower scores indicating poorer performance in the evaluated domains and reduced HRQoL. The questionnaire was administered in an interview format by a trained physical therapist.

#### 2.3.2 Codes and Qualifiers of the International Classification of Functioning, Disability and Health

The linking process between the SF‐36 and the ICF was entirely developed by Bernardelli et al. (2021). In their study, two evaluators independently and blindly performed the linkage process, identifying the main concept addressed in each SF‐36 item and subsequently linking each item to the relevant ICF categories and codes, according to the linking rules developed by Cieza et al. (2019). The resulting linkage is presented in Table 1.

**TABLE 1:** Relationship between SF‐36 domains and ICF categories.

SF‐36 domain	ICF code	Description of ICF category
Physical functioning	d	Activity and participation component
	d4	Mobility
	d430	Lifting and carrying objects
	d4101	Squatting
	d4102	Kneeling
	d4105	Bending
	d4300	Lifting
	d4500	Walking short distances
	d4501	Walking long distances
	d4551	Climbing
	d4552	Running
	d5	Self‐care
	d510	Washing oneself
	d540	Dressing
	d6402	Cleaning living area
	d6403	Using household appliances
	d9201	Sports
Role physical	d230	Carrying out daily routine
	d850	Remunerative employment
Bodily pain	b280	Sensation of pain
General health	NC	NC
Vitality	b152	Emotional functions
	b1263	Psychic stability
	b1300	Energy level
Social functioning	d750	Informal social relationships
	d9205	Socializing
Role emotional	d230	Carrying out daily routine
	d850	Remunerative employment
Mental health	b152	Emotional functions
	b1265	Optimism
	b1300	Energy level

Abbreviations: ICF, International Classification of Functioning, Disability and Health; NC, Not covered by ICF; SF‐36, *Short‐Form Health Survey*.

Building upon this, SF‐36 domain scores were expressed using ICF qualifiers following the approach proposed by Fréz et al. (2014) (Table 2). The ICF generic qualifier scale ranges from 0.0 to 0.4, where 0.0 indicates no problem, 0.1 a mild problem, 0.2 a moderate problem, 0.3 a severe problem, and 0.4 a complete problem. To apply this method, the median score for each SF‐36 domain was calculated and matched to the corresponding ICF qualifier range presented in Table 2. The resulting qualifier was then assigned to the ICF categories previously linked to that domain by Bernardelli et al. (2021). The present study did not perform an independent linking process.

**TABLE 2:** Relationship between ICF qualifiers and SF‐36 domain scores.

ICF qualifier	Qualitative description	SF‐36 domain score
0.0	No problem	96–100
0.1	Mild problem	76–95
0.2	Moderate problem	51–75
0.3	Severe problem	5–50
0.4	Complete problem	0–4

Abbreviations: ICF, International Classification of Functioning, Disability and Health; SF‐36, *Short‐Form Health Survey*.

### 2.4 Procedures

The assessment was performed by physiotherapists properly trained with both theoretical and practical approaches to the instrument. During the interview, participants completed a questionnaire addressing sociodemographic variables (sex, age, income, marital status, amount of medication used, and schooling). Subsequently, the individuals were evaluated using the SF‐36, and the data were tabulated for analysis and conversion of the scores of each domain into ICF qualifiers according to the calculation proposed in Table 2.

### 2.5 Data Analysis

The Kolmogorov‐Smirnov normality test was used to determine the distribution of the data. Descriptive analysis was performed for the characterization of the sample and the distribution of the measures. Mean and standard deviation values were used for quantitative variables with normal distribution. Categorical variables were expressed as frequencies. Descriptive statistics were used to obtain the median score in the sample studied on each SF‐36 domain, followed by the conversion of the ICF qualifier as described in Table 2. Inferential analyses examining relationships between demographic or clinical variables and ICF qualifier classifications were not performed, given the small sample size and the exclusively descriptive objectives of this study.

## 3 Results

Fifty‐six older adults were recruited for the present study; four were excluded due to cognitive impairment assessed by the MMSE. Therefore, the final sample consisted of 52 older adults. Table 3 displays the demographic and social characteristics of participants. The mean age of the participants was 71.6 ± 7.0, only 7.7% over 80 years of age. 92.3% of the sample was composed of women, 46.1% was widowed, 59.6% had a monthly income of 2–3 times the minimum wage and 65.4% met the criteria for polypharmacy (use of five or more medications).

**TABLE 3:** Clinical and demographic characteristics of participants.

Variable	*n* = 52
Sex—*n* (%)	
Men	4 (7.7)
Women	48 (92.3)
Age—mean ± SD	
Overall	71.6 ± 7.0
60–79 years	48 (92.3)
≥ 80 years	4 (7.7)
Marital status—*n* (%)	
Single	18 (34.6)
Married	7 (13.5)
Divorced	3 (5.8)
Widowed	24 (46.1)
Schooling—*n* (%)	
≤ 4 years	7 (13.5)
5–8 years	26 (50.0)
≥ 9 years	19 (36.5)
Medication use—*n* (%)	
No medication	6 (11.5)
1–4 medications	12 (23.1)
≥ 5 medications (polypharmacy)	34 (65.4)
Monthly income—*n* (%)	
1 × minimum wage	16 (30.8)
2–3 × minimum wage	31 (59.6)
> 3 × minimum wage	5 (9.6)

*Note:* Data expressed as absolute and relative frequency and mean ± standard deviation. Monthly minimum wage: Brazilian federal reference value at the time of data collection.

Table 4 displays SF‐36 domains and the relationship between ICF codes, SF‐36 domain scores and ICF qualifiers; median values are used due to the nonparametric distribution of the SF‐36 data. The SF‐36 domains “Bodily pain”, “General Health” and “Vitality” presented a moderate classification [0.2] with the ICF qualifiers. “Physical functioning”, “Social functioning” and “Mental health” domains presented mild problems [0.1]. “Role physical” and “Role emotional” did not present any problem.

**TABLE 4:** Median SF‐36 domain scores and corresponding ICF qualifier classifications.

SF‐36 domain	SF‐36 score^a^ median (25%–75%)	ICF qualifier
Bodily pain	63 (51–84)	0.2 moderate problem
General health	73.5 (61.5–85.5)	0.2 moderate problem
Vitality	70 (58.75–85)	0.2 moderate problem
Physical functioning	80 (63.75–95)	0.1 mild problem
Social functioning	87.5 (62.5–100)	0.1 mild problem
Mental health	76 (66–88)	0.1 mild problem
Role physical	100 (68.75–100)	0.0 No problem
Role emotional	100 (47.9–100)	0.0 No problem

*Note:* Specific ICF categories linked to each SF‐36 domain are presented in Table 1. The scoring criteria used to assign ICF qualifiers are presented in Table 2.

Abbreviations: ICF, International Classification of Functioning, Disability and Health; SF‐36, Short‐Form Health Survey.

^a^ Data expressed as median and interquartile range (25%–75%).

## 4 Discussion

This study applied a previously proposed conversion method to describe HRQoL domain scores from the SF‐36 using ICF qualifiers in community‐dwelling older adults attending primary care services. This application classified 27 ICF categories across eight SF‐36 domains in a sample of 52 older adults. Impairments were generally classified as mild to moderate, whereas no impairment was identified in the “Role Physical” and “Role Emotional” domains. The resulting qualifier classifications allow SF‐36 outcomes to be expressed using the standardized terminology of the ICF. For example, a median SF‐36 “Bodily Pain” score of 63 corresponds to qualifier 0.2 (moderate problem) and is linked to ICF category b280 (Sensation of pain). Rather than being represented solely as a numerical score, the result can also be described according to the severity levels defined by the ICF generic qualifier scale. This descriptive approach offers a structured way of representing HRQoL outcomes within a functioning‐based framework, consistent with the ICF's standardized terminology.

In the sample composition, a decline in the number of individuals over 80 years old was observed, consistent with findings from both Brazilian and global studies (Lima et al. 2009; Krawczyk‐Suszek and Kleinrok 2022). Additionally, there was a notable discrepancy in gender distribution, with women comprising 92.3% of the sample and men only 7.7%. This sex imbalance may have influenced domain‐level scores, particularly those related to “Bodily Pain” and “Vitality”, which exhibit known sex‐related differences in older adults (Krawczyk‐Suszek and Kleinrok 2022). Future studies should recruit more balanced samples or conduct sex‐stratified analyses to examine potential differential effects on ICF qualifier classification.

Moderate impairment was observed in the “Bodily Pain” domain of the SF‐36, likely reflecting the high prevalence of chronic pain among older adults, which affects 30%–50% of this population, varying by region and country (Patel et al. 2013; Bernfort et al. 2015). Chronic pain is strongly associated with reduced HRQoL, with more severe impacts in cases of intense and persistent pain, particularly in older adults (Bernfort et al. 2015). Additionally, it can significantly hinder the performance of daily activities, further compromising functional independence (Patel et al. 2013; Cedraschi et al. 2018). These findings help explain the moderate impairment observed in this domain and align with the results reported by Krawczyk‐Suszek and Kleinrok (2022).

The moderate impairment observed in the “Vitality” domain of the SF‐36 underscores the importance of evaluating this dimension, as vitality is a significant predictor of declines in intrinsic capacity (Bautmans et al. 2022), a key determinant in the aging process, as defined by the WHO. Notably, a similarity was found between the ICF codes linked to the “Vitality” and “Mental Health” domains, with both sharing the codes “b152 Emotional Functions” and “b1300 Energy Level.” Although the SF‐36 scores in both domains were relatively similar (“Vitality” at 70% and “Mental Health” at 76%), the qualifiers differed due to the percentage of impairment observed, as defined by ICF criteria. This difference in qualifiers is likely related to the distinct concepts involved in each domain, with the “Vitality” domain encompassing the concept of “b1263 Psychic Stability,” while the “Mental Health” domain includes “b1265 Optimism.”

The “Physical Functioning,” “Social Functioning,” and “Mental Health” domains exhibited mild impairment. The median scores for these domains observed in the present study fall within the confidence intervals reported by Lima et al. (2009), who investigated HRQoL in Brazilian older adults with demographic characteristics similar to those of our sample, particularly low‐income, low‐education women. Consequently, these findings support and align with the results of Lima et al. (2009).

The observation that the “Role Physical” and “Role Emotional” domains exhibited a median score of 100 can be attributed to several factors related to the sample of community‐dwelling older adults. This population generally exhibits better overall health and functional status compared with individuals residing in institutional settings or experiencing more severe health conditions. Furthermore, community living provides greater opportunities for physical and social activities, which are positively correlated with enhanced quality of life and a diminished impact of physical and emotional limitations. The autonomy and independence commonly associated with community living may also contribute to a more favorable perception of their well‐being.

Although the “General Health” domain does not have a specific ICF code, it also showed moderate impairment and remains a significant concept for assessing health and providing longitudinal care to the older adults. Even in cases where no ICF code could be assigned, as observed with this domain, the qualifier scale still allows the severity of the self‐reported health perception to be communicated in standardized terms, preserving interpretive consistency across domains.

The interpretation of HRQoL assessment results using ICF qualifiers enables a structured description of the severity of problems across different HRQoL domains by translating numerical scores into ICF qualifiers (Santos et al. 2022). In this context, expressing SF‐36 outcomes through ICF qualifiers makes it possible to identify which domains are associated with mild, moderate, or severe problems according to the ICF qualifier scale. For example, rather than documenting only that an older adult scored 63 on the SF‐36 “Bodily Pain” subscale, a physiotherapist may additionally record a qualifier of 0.2 (moderate problem) for ICF category b280 (Sensation of pain) in the clinical record. Similarly, a Social Functioning score yielding qualifier 0.1 (mild problem) for d750 (Informal social relationships) could be explicitly documented in the physiotherapy record to highlight participation‐related concerns that might otherwise remain less visible in numerical scores alone. Thus, ICF qualifiers do not replace SF‐36 scores but may be documented alongside them, providing a standardized description of problem severity based on the ICF framework.

However, it is important to note that the use of sample‐level median scores to derive ICF qualifiers represents a population‐level characterization of functioning. This approach does not capture within‐sample heterogeneity, as individual participants may present substantially different qualifier profiles from those reflected by the group median. Although the present study focused on describing group‐level outcomes, the conversion method proposed by Fréz et al. (2014) is intended for individual‐level application. Future studies should explore the assignment of ICF qualifiers at the individual level, which may provide a more detailed characterization of functioning and permit the examination of variability across participants.

Despite the relevant findings, some limitations should be acknowledged. First, participants were recruited through convenience sampling from a primary healthcare service. Consequently, the sample may not be representative of all community‐dwelling older adults, particularly those who do not regularly access healthcare services. This may have influenced the observed HRQoL profile, including the absence of impairment in the “Role Physical” and “Role Emotional” domains. Therefore, the findings should be interpreted as applicable primarily to older adults receiving primary care in a Brazilian metropolitan context. Second, ICF qualifiers were assigned based on the median score of each SF‐36 domain rather than on individual participant profiles. As a result, the reported qualifiers represent a group‐level characterization of functioning and do not capture within‐sample variability. Although the conversion method proposed by Fréz et al. (2014) was originally conceived for individual‐level application, the use of median scores was considered appropriate for the descriptive objectives of the present study. Third, the cutoff values used to convert SF‐36 domain scores into ICF qualifiers were derived from the approach proposed by Fréz et al. (2014) and have not been independently validated in community‐dwelling older adults. Future studies should examine the measurement properties of these thresholds in geriatric populations and determine whether alternative cutoff values are warranted. Finally, and importantly, no inferential analyses were conducted in this study. Given the small sample size (*n* = 52) and the exclusively descriptive cross‐sectional design, statistical analyses examining associations or relationships between demographic variables (e.g., age, sex, income, polypharmacy) and ICF qualifier classifications were neither planned nor performed. Readers should therefore not interpret the reported qualifier classifications as reflecting differential patterns across subgroups. Future studies with larger and more representative samples and appropriate inferential designs should investigate whether sociodemographic and clinical variables are associated with different patterns of qualifier classification across SF‐36 domains.

## 5 Conclusion

This study applied an existing SF‐36‐to‐ICF conversion method in a sample of community‐dwelling older adults attending primary care services in a middle‐income country. The approach enabled HRQoL outcomes to be expressed according to the ICF qualifier structure alongside conventional SF‐36 scores. Rather than relying exclusively on numerical values, this method provides an additional description of the domains most affected.

## 6 Implications of Physiotherapy Practice

— Converting SF‐36 scores into ICF qualifiers allows HRQoL results to be interpreted in terms of problem severity rather than as isolated numerical values.
— The use of ICF qualifiers helps identify domains associated with mild, moderate, or severe problems, providing an additional description of the domains most affected.
— Because the approach is based on the widely used SF‐36, it has the potential to be applied in contexts where this instrument is already routinely used.

## Author Contributions

Maik Berber Freitas was responsible for the conception and design of the study, methodology, data collection, formal analysis, data interpretation, manuscript writing, and critical revision of the content. Gustavo Pietracatelli Janiziello contributed to statistical analysis, table and figure preparation, and critical revision of the content. Wallace Pereira Silva, Léia Cordeiro de Oliveira, Vívian Elaine Alflen Soares, Ariadne Cardoso da Silva, Bibiana Caldeira Monteiro, and Cid André Fidelis de Paula Gomes participated in data interpretation and provided critical revision of the manuscript. Soraia Micaela Silva coordinated the overall study, conceived the project, contributed to data analysis, provided critical revision of the manuscript, and collaborated in manuscript writing.

## Funding

This study was partially supported by Coordenação de Aperfeiçoamento de Pessoal de Nível Superior (CAPES)—Brazilian Federal Agency for Support and Evaluation of Graduate Education of the Ministry of Education, finance code 001.

## Ethics Statement

This study was approved by the Human Research Ethics Committee of Nove de Julho University, São Paulo, Brazil, in 2021 (approval number: 093645/2021).

## Consent

Consent was obtained from all individual participants included in the study.

## Conflicts of Interest

The authors declare no conflicts of interest.

## Data Availability

The data sets generated and/or analyzed during the current study are available from the corresponding author upon reasonable request.

## References

[pri70271-bib-0001] Bautmans, I. , V. Knoop , A. Thiyagarajan , et al. 2022. “WHO Working Definition of Vitality Capacity for Healthy Longevity Monitoring.” Lancet Healthy Longevity 3, no. 11: e789–e796. 10.1016/S2666-7568(22)00200-8. 36356628 PMC9640935

[pri70271-bib-0002] Bernardelli, R. S. , B. C. Santos , K. O. Scharan , K. P. Corrêa , M. I. B. Silveira , and A. D. L. Moser . 2021. “Application of the Refinements of ICF Linking Rules to the Visual Analogue Scale, Roland Morris Questionnaire and SF‐36.” Ciência & Saúde Coletiva 26, no. 3: 1137–1152. 10.1590/1413-81232021263.03502019. 33729366

[pri70271-bib-0003] Bernfort, L. , B. Gerdle , M. Rahmqvist , M. Husberg , and L. Å. Levin . 2015. “Severity of Chronic Pain in an Elderly Population in Sweden—Impact on Costs and Quality of Life.” Pain 156, no. 3: 521–527. 10.1097/01.j.pain.0000460336.31600.01. 25599240

[pri70271-bib-0004] Brasil . 2015. Lei n° 13.146, de 6 de julho de 2015: Institui a Lei Brasileira de Inclusão da Pessoa com Deficiência (Estatuto da Pessoa com Deficiência). Diário Oficial da União.

[pri70271-bib-0006] Cedraschi, C. , C. Ludwig , A. F. Allaz , F. R. Herrmann , and C. Luthy . 2018. “Pain and Health‐Related Quality of Life (HRQoL): A National Observational Study in Community‐Dwelling Older Adults.” European Geriatric Medicine 9, no. 6: 881–889. 10.1007/s41999-018-0114-7. 34674476

[pri70271-bib-0007] Ciconelli, R. M. , M. B. Ferraz , W. Santos , I. Meinão , and M. R. Quaresma . 1999. “Tradução para língua portuguesa e validação do questionário genérico de avaliação de qualidade de vida SF‐36.” Revista Brasileira de Reumatologia 39: 143–150.

[pri70271-bib-0008] Cieza, A. 2019. “Rehabilitation: The Health Strategy of the 21st Century, Really?” Archives of Physical Medicine and Rehabilitation 100, no. 11: 2212–2214. 10.1016/j.apmr.2019.05.019. 31128114

[pri70271-bib-0009] Cieza, A. , N. Fayed , J. Bickenbach , and B. Prodinger . 2019. “Refinements of the ICF Linking Rules to Strengthen Their Potential for Establishing Comparability of Health Information.” Disability and Rehabilitation 41, no. 5: 574–583. 10.3109/09638288.2016.1145258. 26984720

[pri70271-bib-0010] Cieza, A. , and G. Stucki . 2005. “Content Comparison of Health‐Related Quality of Life (HRQoL) Instruments Based on the International Classification of Functioning, Disability and Health (ICF).” Quality of Life Research 14, no. 5: 1225–1237. 10.1007/s11136-004-4773-0. 16047499

[pri70271-bib-0011] dos Santos, H. M. , L. C. de Oliveira , S. R. Bonifácio , et al. 2022. “Use of the International Classification of Functioning, Disability and Health (ICF) to Expand and Standardize the Assessment of Quality‐of‐Life Following a Stroke: Proposal for the Use of Codes and Qualifiers.” Disability and Rehabilitation 44, no. 24: 7449–7454. 10.1080/09638288.2021.1995055. 34752176

[pri70271-bib-0012] Franchignoni, F. , and F. Salaffi . 2003. Quality of Life Assessment in Rehabilitation Medicine. European Journal of Physical and Rehabilitation Medicine.

[pri70271-bib-0013] Fréz, A. R. , A. A. Abdallah , C. Riedi , J. Galindo , J. A. Ruaro , and S. C. Ribeiro . 2014. “Proposed Use of the International Classification of Functioning, Disability and Health to Evaluate Quality of Life After an Amputation.” Fisioterapia em Movimento 27, no. 1: 49–56. 10.1590/0103-5150.027.001.AO05.

[pri70271-bib-0014] Gimigliano, F. , and S. Negrini . 2017. “The World Health Organization ‘Rehabilitation 2030: A Call for Action’.” European Journal of Physical and Rehabilitation Medicine 53, no. 2. 10.23736/s1973-9087.17.04746-3. 28382807

[pri70271-bib-0015] Krawczyk‐Suszek, M. , and A. Kleinrok . 2022. “Health‐Related Quality of Life (HRQoL) of People Over 65 Years of Age.” International Journal of Environmental Research and Public Health 19, no. 2: 625. 10.3390/ijerph19020625. 35055448 PMC8776108

[pri70271-bib-0016] Laguardia, J. , M. R. Campos , C. M. Travassos , A. L. Najar , L. A. Anjos , and M. M. Vasconcellos . 2011. “Psychometric Evaluation of the SF‐36 (v.2) Questionnaire in a Probability Sample of Brazilian Households: Results of the Survey Pesquisa Dimensões Sociais das Desigualdades (PDSD), Brazil, 2008.” Health and Quality of Life Outcomes 9, no. 1: 61. 10.1186/1477-7525-9-61. 21812986 PMC3162522

[pri70271-bib-0018] Lima, M. G. , M. B. A. Barros , C. L. G. César , M. Goldbaum , L. Carandina , and R. M. Ciconelli . 2009. “Health‐Related Quality of Life Among the Elderly: A Population‐Based Study Using SF‐36 Survey.” Cadernos de Saúde Pública 25, no. 10: 2159–2167. 10.1590/S0102-311X2009001000007. 19851616

[pri70271-bib-0019] Lins, L. , and F. M. Carvalho . 2016. “SF‐36 Total Score as a Single Measure of Health‐Related Quality of Life: Scoping Review.” SAGE Open Medicine 4: 2050312116671725. 10.1177/2050312116671725. 27757230 PMC5052926

[pri70271-bib-0020] Patel, K. V. , J. M. Guralnik , E. J. Dansie , and D. C. Turk . 2013. “Prevalence and Impact of Pain Among Older Adults in the United States: Findings From the 2011 National Health and Aging Trends Study.” Pain 154, no. 12: 2649–2657. 10.1016/j.pain.2013.07.029. 24287107 PMC3843850

[pri70271-bib-0021] Peres, P. A. T. , C. M. Buchalla , and S. M. Silva . 2018. “Aspectos da sobrecarga e qualidade de vida de cuidadores de pacientes hospitalizados: Uma análise baseada na Classificação Internacional de Funcionalidade, Incapacidade e Saúde (CIF).” Revista Brasileira de Saúde Ocupacional 43. 10.1590/2317-6369000013617.

[pri70271-bib-0022] Silva, S. M. , J. C. F. Corrêa , G. S. Pereira , and F. I. Corrêa . 2019. “Social Participation Following a Stroke: An Assessment in Accordance With the International Classification of Functioning, Disability and Health.” Disability and Rehabilitation. 10.1080/09638288.2017.1433428. 29233002

[pri70271-bib-0023] World Health Organization . 2001. International Classification of Functioning, Disability and Health. World Health Organization.

[pri70271-bib-0024] World Health Organization . 2020. Decade of Healthy Ageing: Baseline Report. World Health Organization. Licence: CC BY‐NC‐SA 3.0 IGO.

[pri70271-bib-0025] World Health Organization . 2023a. Landmark Resolution on Strengthening Rehabilitation in Health Systems. https://www.who.int/news/item/27‐05‐2023‐landmark‐resolution‐on‐strengthening‐rehabilitation‐in‐health‐systems.

[pri70271-bib-0026] World Health Organization . 2023b. Progress Report on the United Nations Decade of Healthy Ageing, 2021–2023. World Health Organization. Licence: CC BY‐NC‐SA 3.0 IGO.

